# Intérêts de l'examen du fond d’œil en pratique de ville: bilan de 438 cas

**DOI:** 10.11604/pamj.2015.20.363.6629

**Published:** 2015-04-14

**Authors:** Jean Wenceslas Diallo, Nonfounikoun Méda, Somnoma Jean Baptiste Tougouma, Ahgbatouhabéba Ahnoux-Zabsonré, Rosane Yé, Pierre Djiguimdé, Jérôme Sanou, Paté Sankara, Gertrude Méda, Mariam Dolo

**Affiliations:** 1 Centre Hospitalier Universitaire de Bobo Dioulasso, Bobo Dioulasso, Burkina Faso; 2 Centre Hospitalier Universitaire Yalgado Ouédraogo, Ouagadougou, Burkina Faso

**Keywords:** Fond d´œil, rétinopathie hypertensive, rétinopathie diabétique, rétinopathie drépanocytaire, Fondus, hypertensive retinopathy, diabetic retinopathy, sickle cell retinopathy

## Abstract

L'examen du fond d’œil fait partie du bilan de nombreuses maladies générales en dehors de l'ophtalmologie. L'objectif de notre travail était d’étudier les aspects cliniques et épidémiologiques des patients adressés pour un fond d’œil afin de montrer l'intérêtde cet examen. Il s'est agi d'une étude rétrospective descriptive des examens du fond d’œil durant la période de janvier 2011 à décembre 2013 dans un cabinet d'ophtalmologie d'une polyclinique à Bobo Dioulasso. Au cours de la période étudiée, 5942 consultations ont été enregistrées, dont 438 pour fond d’œil soit 7,37%. Il y avait 225 hommes et 213 femmes soit un sex ratio de 1,056. La tranche d’âge 40-59 ans représentait 54%. La fréquence des principaux motifs de la demande était l'hypertension artérielle 43,15% (N=189), le diabète 39,04% (N=171), l'association HTA et diabète 10,27% (N=45), et la drépanocytose 7,53% (N=33). Le fond d’œil était anormal chez 175 patients soit 36,23%. La rétinopathie hypertensive était retrouvée dans 42,73% des cas, la rétinopathie diabétique 25,92%, et la rétinopathie drépanocytaire 7,53%. L'examen du fond d’œil en médecine de ville présente un intérêt majeur, et permet de retrouver des anomalies chez plus d'un tiers des patients.

## Introduction

Bobo-Dioulasso est le chef-lieu de la région des Haut-bassins et représente la 2^ème^ ville du Burkina Faso. Dans la ville il existe 5 structures publiques dont le Centre Hospitalier Universitaire Sourô Sanou (CHUSS), et 3 cabinets privés pour les soins oculaires. La pathologie oculaire dans la ville est cosmopolite. De nombreuses affections générales ont un retentissement sur le fond d’œil. L'hypertension artérielle, le diabète, la drépanocytose et les affections inflammatoires systémiques sont les plus courantes. L'examen clinique en ophtalmologique en particulier l'examen du fond d’œil est très souvent utile pour faire le bilan de retentissement de ces affections générales. C'est un temps important qui permet l'analyse macroscopique de la rétine en particulier. Le but de notre travail a été d’étudier les aspects épidémiologiques et cliniques des patients adressés pour un examen du fond d’œil, rappeler l'intérêt du fond d’œil et de contribuer à la prise en charge multidisciplinaire deaffections générales.

## Méthodes

Le champ de notre étude a été la ville de Bobo Dioulasso et le cadre une polyclinique privée de la place de Bobo Dioulasso, située dans le centre-ville. Nous avons réalisé une étude rétrospective descriptive de janvier 2011 à décembre 2013. La population de notre étude était constituée des patients adressés pour un examen du fond d’œil au cours de cette période. Les données ont été recueillies à partir des dossiers des patients, et des registres de consultation. Nous avons décrit les variables sociodémographiques, les antécédents cliniques, les motifs de la demande du fond d’œil, les résultats cliniques de l'examen du fond d’œil. Nous avons utilisé la classification de Kirkendall [1] pour la rétinopathie hypertensive, celle de l'association de langue française pour l’étude du diabète et des maladies métaboliques (ALFEDIAM) [2] pour la rétinopathie diabétique (RD), et enfin la classification de Goldberg [3] pour la rétinopathie drépanocytaire.

La saisie des données a été réalisée à l'aide du logiciel EPI-Info version 7 et l'analyse a été faite grâce au logiciel Stata. Les tableaux ont été faits grâce à Excel 2007. Pour les variables quantitatives indépendantes, les moyennes accompagnées des écart-type ont été utilisées et pour les variables qualitatives nous avons utilisés les fréquences relatives. Le test de Chi2 de Pearson ou le test de Fisher ont été utilisés pour la comparaison des proportions.

## Résultats

Au cours de la période de notre étude, 5942 consultations ont été faite en ophtalmologie. Nous avons inclus 438 patients adressés pour un examen du fond d’œil pendant la période, ce qui représentait 7,37% des consultations. L’âge moyen était de 43 ans (écart type de 11,02) avec des extrêmes de 18 et 72 ans. La tranche d’âge 40-59 ans était la plus représentée avec 243 cas soit 54%. Il y avait 225 hommes et 213 femmes soit un sex-ratio de 1,05. Les motifs de la demande de l'examen ont été par ordre de fréquence l'hypertension artérielle (HTA) 189 cas soit 43,15%, le diabète 171 cas 39,04%, les deux associés 45 cas 10,27% et la drépanocytose 33 cas 7,53%. Sur l'ensemble des 438 patients inclus, le fond d’œil était normal dans 308 cas soit 63,76%, et anormal dans 130 cas soit 36,33%. Chez les patients hypertendus, le fond d’œil était anormal dans 100 cas soit 42,74%, et la rétinopathie hypertensive stade I de Kirkendall était la plus fréquente avec 85 cas soit 36,32% (Tableau 1). Chez les diabétiques, une rétinopathie était présente dans 56 cas soit 25,92%, et la rétinopathie diabétique non proliférante était prédominante avec 53 cas soit 24,53% (Tableau 2). Parmi les patients examinés pour drépanocytose le fond d’œil était anormal dans 19 cas soit 57,57% et la rétinopathie drépanocytaire stade III de Goldberg était prédominante 21,21% (Tableau 3).

**Tableau 1:** Répartition des patients hypertendus selon la classification de Kirkendall

Stade	Nombre n=189	Pourcentage
Rétinopathie hypertensive stade I	85	36,32%
Rétinopathie hypertensive stade II	13	5,55%
Rétinopathie hypertensive stade III	2	0,85%

**Tableau 2:** Répartition des patients diabétiques selon la classification de l'ALFEDIAM

Stade	Nombre n= 171	Pourcentage
Rétinopathie diabétique non proliférante RDNP	53	24,53%
Minime	23	41,07%
Modérée	29	51,78%
Sévère	4	7,14%
Rétinopathie diabétique proliférante	3	1,38%
Maculopathie diabétique	8	3,70%

**Tableau 3:** Répartition des patients drépanocytaires selon la classification de Goldberg

Stade	Nombre (n=19)	Pourcentage
Rétinopathie drépanocytaire stade 1	3	9,09%
Rétinopathie drépanocytaire stade 2	5	15,15%
Rétinopathie drépanocytaire stade 3	7	21,21%
Rétinopathie drépanocytaire stade 4	4	12,12%

## Discussion

Notre travail présente les limites liées à une étude rétrospective. Une autre limite est le cadre de cette étude qui a été une clinique privée, ce qui peut constituer un biais de sélection des patients. Néanmoins nous pensons que les résultats de ce travail qui a montré que l'examen du fond d’œil représente un peu moins de 10% de la consultation présentent un intérêt pour une discussion. Les principales indications de la demande d'examen du fond d’œil ont été les bilans de retentissement de l'hypertension artérielle, du diabète et de la drépanocytose.

### L'hypertension artérielle

**Figure 1:**
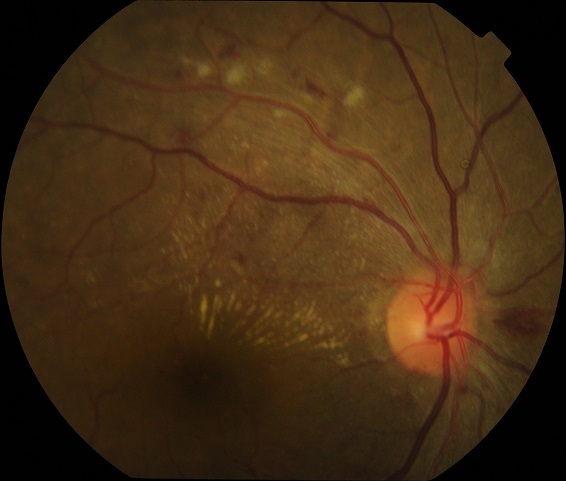
Rétinographie: rétinopathie hypertensive stade 2 de Kirkendall. Noter la dilatation veineuse, les nodules cotonneux, les hémorragies rétiniennes, et les exsudats circinés formant une demi-étoile maculaire

L'HTA affecte 27 à 30%de la population des plus de 20 ans en Afrique Sub Saharienne et concernerait plus de 80 millions d'adultes [4]. Au Burkina Faso, Niakara rapporte une prévalence dans la population générale de 40,2% [5]. La rétinopathie hypertensive est l'ensemble des lésions rétiniennes d'origine vasculaire dues à la vasoconstriction et à la hyalinisation progressive de la paroi artérielle secondaire à l'HTA. Ses manifestations chroniques sont le plussouvent irréversibles, alors que les manifestations aigues sont réversibles [1, 2]. Les signes observés au fond d’œil dans la rétinopathie hypertensive sont le rétrécissement vasculaire diffus ou localisé, les occlusions artériolaires, les nodules cotonneux, les hémorragies rétiniennes superficielles, les exsudats secs souvent maculaires, et l'ischémie choroïdienne (Figure 1) [6].

La rétinopathie hypertensive survient avec l’âge et la sévérité de la tension artérielle [7, 8]. En effetdans la thèse de Yaméogoen milieu hospitalier à Bobo Dioulasso, la tranche âge 51-60 ans représentait 25%, et celle de 61-70 ans 23,10%. Dans notre étude les 40- 59 ans représentaient 55,47%. Le même auteur rapporte 65,3% de cas de rétinopathie hypertensive, ce qui est nettement plus élevé que les 42,74% de notre étude. Cette différence est très probablement due à la population étudiée. En effet les cas hospitaliers sont souvent les plus graves, et les patients arrivent dans des tableaux d'HTA sévère et mal équilibrée évoluant depuis longtemps. Cette gravité s'observe également dans la classification des rétinopathies avec 36,32% de stade 1 de Kirkendall dans notre étude contre 42% de stade 3 de Keith et Wegner pour Yaméogo [7]. La prévalence élevée de rétinopathie hypertensive justifie l'examen du fond d’œil chez le patient hypertendu. Il permet de rechercher les signes d'atteinte aigüe, declasser l'atteinte rétinienne et assurer un meilleur suivi du patient par son cardiologue. Selon Wong et al la rétinopathie hypertensive serait un indicateur de risque de morbidité et de mortalité systémique [8]. Austade de début, la rétinopathie hypertensive est peu associée aux atteintes vasculaires, alors que le stade 2 de Kirkendall est fortement associé aux pathologies vasculo-cérébrales [9]. La rétinopathie hypertensive permet d’établir un pronostic ce qui fait toute l'importance de l'examen du fond d’œil. Aussi selon les recommandations de la Société européenne de cardiologie et la Société européenne d'hypertension artérielle [10], l'examen du FO s'impose dans les cas suivants: une HTA sévère de diagnostic récent: à la recherche d'une rétinopathie hypertensive maligne; une baisse d'acuité visuelle chez un hypertendu: à la recherche d'une occlusion ou d'un œdème; chez un patient hypertendu diabétique: un fond d’œil annuel pour rechercher une rétinopathie diabétique associée; pas d'examen du fond d’œil systématique en cas d'HTA non compliquée et bien équilibrée.

### Le diabète

Le deuxième motif de demande du fond d’œil a été le bilan de diabète. Cette maladie est considérée comme un problème majeur de santé public dans les pays développés et prend des proportions importantes au Burkina Faso. L'estimation 2014 de laprévalence nationale du diabète au Burkinaest de 3,2% de la population [11]. Dans la littérature, une des plus grandes études menées aux Etats Unis sur la rétinopathie diabétique indique que 40% des diabétiques sont porteurs d'une rétinopathie [12]. Au Burkina Faso dans une étude en milieu hospitalier à Ouagadougou Sawadogo rapporte une fréquence de la RD de 37,5% et Méda 43,1% [13, 14]. Ces taux sont beaucoup plus élevés que ce qui est observé dans notre étude avec 25,92%. Là encore nous pensons que la population suivie en milieu hospitalier présente un diabète plus grave ou plus évolué, ce qui pourrait expliquer la différence de nos chiffres.

**Figure 2:**
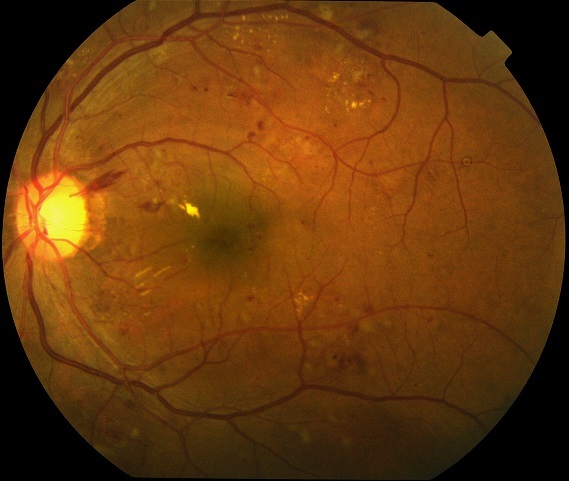
Rétinographie: rétinopathie diabétique non proliférante modérée: nombreux micro anévrysmes, des hémorragies rétiniennes, nodules cotonneux et exsudats secs en temporal supérieur

Les principaux signes de la rétinopathie diabétique (RD) au fond d’œil sont les microanévrysmes, les hémorragies rétiniennes, les nodules cotonneux, les exsudatssecs, les anomalies veineuses, l'occlusion vasculaire, l'ischémie rétinienne, et les néovaisseaux (Figure 2) [2]. Les facteurs de risque identifiés sont l'ancienneté du diabète, le mauvais équilibre glycémique ou tensionnel [12]. Les circonstances de progression de la rétinopathie diabétique sont la grossesse, l'adolescence, la puberté, l’équilibration rapide du diabète, la chirurgie oculaire [12]. Ces circonstances particulières associées aumode d'installation insidieux de la RD justifientla surveillance régulière du fond d’œil du diabétique. Aussi, l'ALFEDIAM préconise les recommandations suivantes. En l'absence de rétinopathie diabétique, ou en cas de RD minime, un examen par an. En cas de RD plus grave, une surveillance ophtalmologique tous les 4 à 6 mois peut être nécessaire. Une surveillance rapprochée dans les cas de puberté etd'adolescence, grossesse, normalisation rapide de la glycémie, chirurgie de la cataracte, et de décompensation d'une HTA ou rénale [2].

### La drépanocytose

**Figure 3:**
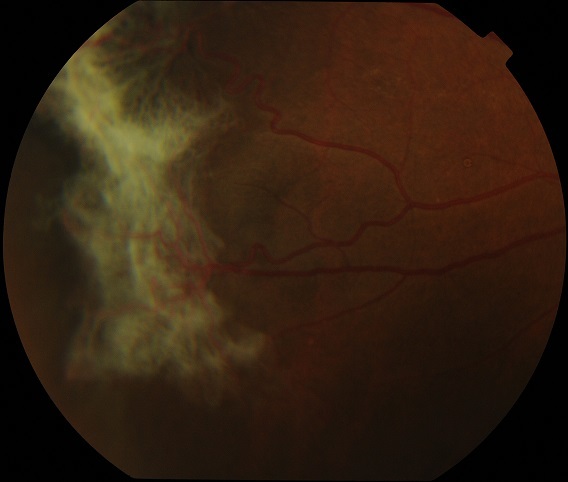
Rétinographie: rétinopathie drépanocytaire proliférante stade 3 de Goldberg: de gros bouquets néo vasculaires formant un aspect de « see fan » en rétine périphérique

Une autre affection qui a justifié la demande d'examen du fond d’œil a été la drépanocytose. Il s'agit de la maladie héréditaire la plus répandue dans le monde et surtout en Afrique. Au Burkina Faso, les syndromes drépanocytaires majeurs (SS et SC) touchent près de 2% des nouveau-nés avec une incidence de 1 sur 57/an [15]. La rétinopathie drépanocytaire est l'ensemble des anomalies vasculaires rétiniennes secondaires aux occlusions vasculaires. Les manifestations au fond d’œil sont essentiellement les occlusions vasculaires, l'ischémie rétinienne, les anastomoses vasculaires, la néovascularisationprérétinienne, et ses complications qui sont l'hémorragie intra vitréenne et le décollement de rétine (Figure 3). La prévalence de la rétinopathie drépanocytaire est variable en Afrique sub-saharienne. En effet Ballo au Togo rapporte 57%, alors que Traoré au Mali trouve une prévalence plus élevée avec 68% [16, 17]. Au Burkina Diallo rapporte 49% de cas de rétinopathie dans un centre médical et Méda en milieu hospitalier rapporte16,5% de cas de rétinopathie drépanocytaire proliférante [18, 19]. Toutes les études montrent qu'elle est plus élevée chez les hétérozygotes composites SC, et elle survient avec l’âge [16, 18, 20–22]. L’évolution vers des complications à savoir l'hémorragie intra vitréenne, et le décollement de la rétine d'une part et l'efficacité du traitement préventif par photo coagulation laser suggèrent que le fond d’œil soit régulièrement fait chez les patients présentant un syndrome drépanocytaire majeur [22]. Ainsi le 1^er^ examen peut être pratiqué chez les sujets SC à partir de 8 ans, à l'adolescence puis annuellement. En présence de rétinopathie, la surveillance est fonction de la gravité. Chez les sujets SS un FO à l'adolescence puis annuellement.

## Conclusion

L'examen du fond d’œil en médecine de ville représente une activité importante qui représente 7,37% de l'ensemble des consultations. Il permet de mettre en évidence dans plus d'un tiers des cas une anomalie. La sévérité des cas semble moins importante que dans la population hospitalière. Dans notre contexte de travail, l'hypertension artérielle, le diabète et la drépanocytose ont été les principaux motifs de demande. L'examen du fond d’œil doit être plus souvent demandé par les praticiens afin d'améliorer la qualité de la prise en charge multidisciplinaire de certaines affections générales.

## References

[1] Walsh JB (1982). Hypertensive retinopathy: description, classification, and prognosis. Ophthalmology..

[2] Lecleire-Collet A, Erginay A, Angioi-Duprez K, Deb-Joardar N, Gain P, Massin P Classification simplifiée de la rétinopathie diabétique adaptée au dépistage par photographies du fond d’œil. /data/revues/01815512/00300007/674/ (Internet). http://www.em-consulte.com/en/article/132886.

[3] Goldberg MF (1971). Classification and pathogenesis of proliferative sickle retinopathy. Am J Ophthalmol..

[4] Fourcade L, Paule P, Mafart B (2007). Arterial hypertension in sub-Saharan Africa: Update and perspectives. Médecine Trop Rev Corps Santé Colon..

[5] Niakara A, Fournet F, Gary J, Harang M, Nébié LVA, Salem G (2007). Hypertension, urbanization, social and spatial disparities: a cross-sectional population-based survey in a West African urban environment (Ouagadougou, Burkina Faso). Trans R Soc Trop Med Hyg..

[6] Wong TY, Klein R, Sharrett AR, Manolio TA, Hubbard LD, Marino EK (2003). The prevalence and risk factors of retinal microvascular abnormalities in older persons: The Cardiovascular Health Study. Ophthalmology..

[7] Yaméogo AA (1997). Etude du profil épidémiologique, clinique et évolutif de l'hypertension artérielle en milieu hospitalier de Bobo Dioulasso: thèse de médecine.

[8] Wong TY, McIntosh R (2005). Hypertensive retinopathy signs as risk indicators of cardiovascular morbidity and mortality. Br Med Bull..

[9] Wong TY, McIntosh R (2005). Systemic associations of retinal microvascular signs: a review of recent population-based studies. Ophthalmic Physiol Opt J Br Coll Ophthalmic Opt Optom..

[10] Recommandations 2007 ESH/ESC (2007). 2007 Guidelines for the management of Arterial Hypertension: The Task for the management of Arterial Hypertension of the European Society of Hypertension (ESH) and the European Society of Cardiology (ESC). J Hypertens.

[11] http://www.idf.org/sites/default/files/Atlas-poster-2014_FR.pdf.

[12] (1991). Early Treatment Diabetic Retinopathy Study design and baseline patient characteristics. ETDRS report number 7. Ophthalmology.

[13] Sawadogo N (1997). Etude des aspects épidémiologiques et cliniques des principales complications oculaires au cours du diabète sucre au centre hospitalier national YALGADO OUEDRAOGO.

[14] Méda N, Tiono H, Ahnoux-Zabsonre A, Djiguimdé W, Nabaloum Y, Drabo J (2010). La rétinopathie diabétique au Burkina Faso: état des lieux. Rev Société Ouest Afr d'Ophtalmologie.

[15] Kafando E, Sawadogo M, Cotton F, Vertongen F, Gulbis BM, Cotton F, Vertongen F, Gulbis B (2005). Le dépistage néonatal de la drépanocytose à Ouagadougou, au Burkina Faso: une étude pilote. J Med-Screen..

[16] Balo KP, Segbena K, Mensah A, Mihluedo H, Bechetoille A (1996). Hémoglobinopathies et rétinopathies au CHU de Lomé. J Fr Ophtalmol..

[17] Traoré J, Boitre JP, Bogoreh IA, Traoré L, Diallo A (2006). Drépanocytose et atteintes rétiniennes: étude de 38 cas à l'Institut d'ophtalmologie tropicale de l'Afrique de l'Ouest de Bamako-(IOTA). Médecine Trop Rev Corps Santé Colon..

[18] Diallo JW, Sanfo O, Blot I, Meda N, Sawadogo P, Ouedraogo A (2009). Étude épidémiologique et facteurs pronostiques de la rétinopathie drépanocytaire à Ouagadougou (Burkina Faso). J Fr Ophtalmol..

[19] Méda N, Djiguimdé W, Nacoulma E, Ouédraogo P, Drabo Y (2012). Manifestations ophtalmologiques au cours des hémoglobinopathies S et C au Centre Hospitalier Universitaire Yalgado Ouédraogo. Sci Tech Sci Santé.

[20] Leveziel N, Lalloum F, Bastuji-Garin S, Binaghi M, Bachir D, Galacteros F (2012). Rétinopathie drépanocytaire: analyse rétrospective portant sur 730 patients suivis dans un centre de référence. J Fr Ophtalmol..

[21] Balo KP, Fany A, Mihluedo H, Djagnikpo PA, Koffi-Gue KB (1997). Atteinte rétinienne au cours de la drépanocytose au Togo: Corrélation entre âge, génotype et rétinopathie. J Fr Ophtalmol..

[22] Morel C Atteinte rétinienne des hémoglobinopathies. /data/revues/01815512/00240009/987/ (Internet). http://www.em-consulte.com/en/article/111682.

